# P01-039 – Autonomic functions in children with FMF

**DOI:** 10.1186/1546-0096-11-S1-A43

**Published:** 2013-11-08

**Authors:** K Fidanci, M Gulgun, A Kilic, C Acikel, E Demirkaya, S Ozen, F Gok

**Affiliations:** 1 Pediatric Cardiology, Hacettepe University, School of Medicine, Ankara, Turkey; 2 FMF Arthritis Vasculitis and Orphan Disease Research Center, Hacettepe University, School of Medicine, Ankara, Turkey; 3 Pediatric Rheumatology, Gulhane Military Medical Faculty, Hacettepe University, School of Medicine, Ankara, Turkey; 4 Pediatric Rheumatology, Hacettepe University, School of Medicine, Ankara, Turkey

## Introduction

Familial Mediterranean Fever (FMF) is an autoinflammatory disorder characterized by recurrent fever associated with inflammation of serous membranes. There is no study reporting the assessment of autonomic functions by using heart rate variability (HRV) in children with FMF. HRV is a practical and reliable method for evaluation of autonomic functions. HRV studies have pointed to the presence of autonomic dysfunctions in many autoinflammatory disorders, possible contributing factors to ventricular tachyarrhythmias and sudden cardiac death in these patients.

## Objectives

In this study, we investigated possible alterations in cardiac autonomic functions and other probable cardiac effects in children with FMF by HRV analyses and conventional echocardiography.

## Methods

In each patient, it was performed twelve lead electrocardiography (ECG) at 25 mm/s (paper speed), 24 h ambulatory electrocardiographic monitorization (AECG), and transthoracic echocardiography by a Siemens Acuson Sequoia C256 cardiac ultrasonographic scanner, with 2.5- to 3.5-MHz transducers.

## Results

Seventy FMF patients and 50 healthy controls were enrolled in the study. It was noted that SDNN (standard deviation of all NN intervals) value was lower in patients with FMF as compared to the control group. Frequency-dependent HRV parameters were similar in both groups. There was no difference in patient and control groups in terms of conventional echocardiographic parameters.

## Conclusion

Studies with larger cohorts and more comprehensive methods are required to assess the presence and consequences of possible autonomic dysfunction in children with FMF.

## Disclosure of interest

None declared.

